# Genome-Wide Prediction of Vaccine Candidates for* Leishmania major*: An Integrated Approach

**DOI:** 10.1155/2015/709216

**Published:** 2015-11-23

**Authors:** Satarudra Prakash Singh, Kriti Roopendra, Bhartendu Nath Mishra

**Affiliations:** ^1^Amity Institute of Biotechnology, Amity University Uttar Pradesh, Lucknow Campus, Lucknow 226028, India; ^2^Institute of Engineering and Technology, U.P. Technical University, Lucknow 226021, India

## Abstract

Despite the wealth of information regarding genetics of the causative parasite and experimental immunology of the cutaneous leishmaniasis, there is currently no licensed vaccine against it. In the current study, a two-level data mining strategy was employed, to screen the* Leishmania major* genome for promising vaccine candidates. First, we screened a set of 25 potential antigens from 8312 protein coding sequences, based on presence of signal peptides, GPI anchors, and consensus antigenicity predictions. Second, we conducted a comprehensive immunogenic analysis of the 25 antigens based on epitopes predicted by NetCTL tool. Interestingly, results revealed that candidate antigen number 1 (LmjF.03.0550) had greater number of potential T cell epitopes, as compared to five well-characterized control antigens (CSP-*Plasmodium falciparum*, M1 and NP-Influenza A virus, core protein-Hepatitis B virus, and PSTA1-*Mycobacterium tuberculosis*). In order to determine an optimal set of epitopes among the highest scoring predicted epitopes, the OptiTope tool was employed for populations susceptible to cutaneous leishmaniasis. The epitope (127SLWSLLAGV) from antigen number 1, found to bind with the most prevalent allele HLA-A⁎0201 (25% frequency in Southwest Asia), was predicted as most immunogenic for all the target populations. Thus, our study reasserts the potential of genome-wide screening of pathogen antigens and epitopes, for identification of promising vaccine candidates.

## 1. Introduction

Leishmaniases are a group of complex diseases caused by protozoan parasites of the genus* Leishmania* and transmitted to humans by hematophagous sandflies [1]. There are at least 20 species of the parasite, which vary according to geographical location and cause a variety of clinical manifestations, ranging from self-limiting cutaneous lesions to potentially fatal infection of the viscera [2, 3]. It is a disease of tropical and subtropical areas, with more than 12 million cases in 88 countries and 2 million new cases annually including 1.5 million cases of cutaneous leishmaniasis (CL) and 0.5 million cases of visceral leishmaniasis (VL). The cutaneous disease is particularly prevalent in Afghanistan, Algeria, Brazil, Iran, Peru, Saudi Arabia, and Syria, accounting for 90% of the global CL burden [4].

Although high-cost chemotherapeutics are available, they show high toxicity and are prone to drug resistance development due to prolonged treatment periods [5]. Despite substantial effort spent in developing effective vaccines, there is currently no licensed vaccine against human leishmaniasis [6]. A large number of proof-of-principle studies have clearly demonstrated that different vaccine formulations, ranging from killed/live-attenuated parasites to recombinant DNA/protein vaccines, can provide significant protection against infection with* Leishmania* spp. in a variety of animal models [7, 8]. However, the efficacy of these prophylactic or therapeutic vaccines remains partial, and it is therefore necessary to develop novel and effective vaccines [1].

In this regard, antigen identification represents the most important roadblock in vaccine development against any pathogen, as it is usually achieved through rather empirical, time-consuming, and labour-intensive* in vivo* and* in vitro* experiments. Efforts have thus been devoted to the development of novel strategies for a more rational and faster identification of antigens among large number of pathogen proteins [9]. In recent development, reverse vaccinology approach can be used to predict those antigens that are most likely to be vaccine candidates using the pathogen genomic sequence [10].

Moreover, the genomic information, which contains the sequences of all known and unknown potential antigens of each pathogen, has enabled the prediction and analysis of the entire repertoire of potential cytotoxic T lymphocytes (CTL) epitopes using bioinformatics tools. This strategy allows the development of vaccines that were previously difficult or impossible to make and can lead to the discovery of unique antigens that may improve existing vaccines [11]. The recent genomic sequence completion of* L. major, L. braziliensis, L. infantum, and L. donovani* and the availability of immunoinformatics tools have opened new opportunities for the identification of novel vaccine targets against CL [9]. Additionally, the presence of genetically stable but highly conserved antigens among most of the species, including antigens with little or no homology to human proteins, offers hope for the development of a single vaccine for multiple disease indications [12].

As depicted in the literature, effective vaccines must invoke a strong response from both T and B cells; therefore, CTL epitope mapping is crucial in any vaccine designing strategy. Many immunoinformatics algorithms and resources have been available to predict T and B cell immune epitopes for peptide based vaccine design and development [13]. Thus, the approach of T cell epitopes prediction and their* in vitro*/*in vivo* validations appeared to be a very powerful strategy in rational antigen identification, particularly for a pathogen with a large genome such as* Leishmania* [9]. Hence, the current study deals with the analysis of* L. major* genome (33.6 Mb), considered to express about 8300 proteins, all of which are potential antigens containing effective CTL epitopes with respect to susceptible population [14].

## 2. Materials and Methods

### 2.1. Retrieval of Proteome Sequence Dataset

The complete proteome of* L. major* (strain Friedlin), consisting of 8312 protein coding sequences, was extracted from database GeneDB [15]. We also retrieved five well-characterized control antigens (CSP-401GLIMVLSFL from* Plasmodium falciparum*, M1-58GILGFVFTL and NP- 265ILRGSVAHK from Influenza A virus, core-141STLPETTVV from Hepatitis B virus, and PSTA1- 41FVVALIPLV from* Mycobacterium tuberculosis*) from database AntigenDB in order to compare and validate the prediction results. These known antigens have been previously tested and verified in various experimental studies and reported as capable of eliciting CTL responses [16].

### 2.2. Methodology Used for Prediction and Characterization of Candidate Antigens/Epitopes

**Figure 1:**
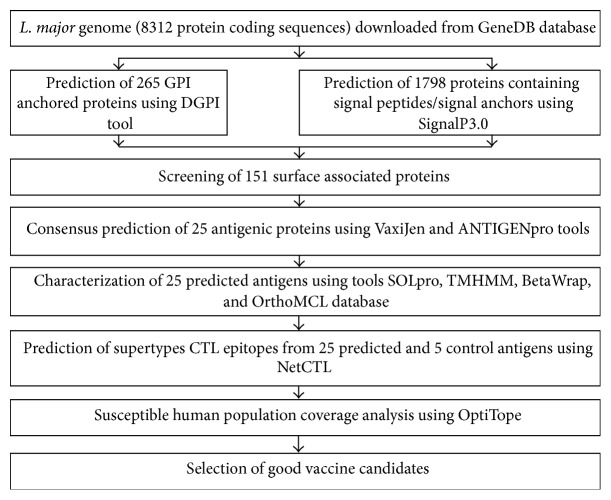
Flowchart depicting the steps adopted for genome-wide screening of potential antigens and their epitopes optimization.

Initially, the* L. major* proteome (8312 proteins) was screened for the presence of both signal peptide and GPI anchors using SignalP [17] and DGPI [18], respectively, and then consensus antigenicity predictions were done using VaxiJen [19] and ANTIGENpro [20] programs. Finally selected candidate antigens were further characterized using TMHMM [21], SCRATCH protein predictor [22], and BetaWrap program [23]. Thereafter, these candidate antigens were searched for potential sequence similarity with other closely related species and human and/or mouse proteins, using OrthoMCL database [24]. Furthermore, CTL epitopes prediction was carried out using NetCTL1.2 [25, 26] tool integrating predictions of proteasomal cleavage, TAP transport efficiency, and 12 MHC class I supertypes' binding. Finally, OptiTope (http://etk.informatik.uni-tuebingen.de/optitope) was used to determine good vaccine epitopes called the optimal set of epitopes from top scoring naturally processed T cell epitopes, for each population susceptible to cutaneous leishmaniasis (Figure 1) [27].

The tool OptiTope requires the following input data from the user: (i) sequences of known/predicted antigens, (ii) a target human population, that is, MHC alleles and corresponding frequency, and (iii) the epitope set to be optimized. The input given by the user is transformed into an optimization problem. If it is feasible, OptiTope will return an optimal set of epitopes along with fraction of immunogenicity contributing to overall immunogenicity. Otherwise, program will propose changes to the user's input that might yield a feasible optimization problem. The information related to MHC alleles frequency in susceptible human populations and geographic areas is retrieved from dbMHC database (http://www.ncbi.nlm.nih.gov/gv/mhc). A good vaccine displays a high overall immunogenicity that means it is capable of inducing potent immunity in a large fraction of the target population including high mutation tolerance as well as a certain degree of allele and antigen coverage. Furthermore, the finally selected epitopes should display a high probability of passing through the natural antigen processing pathway. From all possible epitope combinations, the ones with a maximum overall immunogenicity will be called “optimal” (there may be more than one optimal epitope combination). Hence, the search for an optimal epitope set for an good vaccine can be considered as an optimization problem: out of a given set of epitopes, choose a subset which, out of all subsets meeting the other input requirements, displays maximum overall immunogenicity *I*, which can be derived mathematically (1) as a weighted sum over immunogenicities of epitopes *E* with respect to the given MHC alleles *A*:

(1) $$\begin{array}{c} I=\underset{e\in E}{\sum }\;\underset{a\in A}{\sum }p_{a}\cdot i_{e,a}, \end{array}$$

where *p*_*a*_ is the frequency of allele *a* in the target population and *i*_*e*,*a*_ measure the immunogenicity of epitope *e* when bound to allele *a* (either predicted or experimentally determined).

## 3. Results and Discussion

The present study was divided into two major steps: (i) we utilized the* L*.* major* genome consisting of 8312 protein coding sequences and predicted 25 antigens (Table 1), through successive screening and consensus antigenicity predictions; (ii) we conducted a comprehensive analysis of the epitopes predicted from these 25 candidate antigens (Figure 1). The present strategy is similar to the reverse vaccinology approach adopted by John et al. [28], for identifying common vaccine candidates from* L. major* and* L. infantum* genomes. Additionally, Singh et al. [29, 30] also utilized the similar approach of MHC supertype based epitope identification, as a strategy to mine proteomic data for identification of novel CTL epitopes, in* Plasmodium falciparum*.

### 3.1. Screening of* L. major* Genome for Identification and Characterization of Antigens

The previous studies revealed that surface-exposed proteins such as secretory/outer membrane proteins are ideal targets for vaccine development, with respect to those pathogens against which a strong B cell response (for antibody production) is critical. However, for vaccine development against those pathogens where T cell response is critical, subcellular localization is not an issue since a T cell response could be directed to any protein target [31]. In addition, GPI anchored proteins are abundantly expressed in the infective and intracellular stages of* Trypanosoma cruzi* (another kinetoplastid protozoan) and have been recognized as antigenic targets by both the humoral and cellular immunity [32].

Herein, the entire protein repertoire of* L*.* major*, consisting of 8312 protein coding sequences, was screened for presence of signal peptides and GPI anchors. Out of these, 265 proteins were predicted as GPI anchored proteins, using DGPI tool [18], and 1798 proteins were found to contain signal peptides/signal anchors, using SignalP3.0 tool [17]. However, 151 proteins were predicted to contain both signal peptides/signal anchors and GPI anchors (data not shown). Further screening of these 151 proteins, based on consensus antigenicity predictions using VaxiJen [19] and ANTIGENpro [20] tools, above a predefined threshold of 0.6, provided a set of 27 antigenic proteins (data not shown). Interestingly, three candidate antigens (GeneDB id: LmjF.04.0130, LmjF.04.0140, and LmjF.04.0170) were found to share a high sequence similarity (more than 99.6%) and thus the latter two antigens were excluded from further analysis. Finally, 25 candidate antigens were screened for further characterization as vaccine candidates (Table 1). Protein insolubility has been known to be a major obstacle for many experimental studies. Thus, we used SOLpro tool (of SCRATCH protein predictor [22]) to predict the propensity of a protein to be soluble upon overexpression. Out of the 25 antigens, 8 (numbers 3, 5, 7, 12, 13, 14, 16, and 18) were predicted to be soluble upon overexpression while control antigens M1, core, and PSTA1 were predicted to be insoluble upon overexpression (Table 1).

Similarly, proteins with more than one transmembrane (TM) region have been found to be difficult to clone, express, and purify. Thus, we predicted TM regions using TMHMM web server. Out of 25 predicted antigens, 19 antigens were found to contain less than two; 5 antigens (numbers 6, 11, 12, 13, and 24) were found to contain two each while antigen number 1 was found to contain five TM regions. On the other hand, PSTA1 was found to contain 6 TM regions (Table 1). Through literature analysis, it has also been observed that many bacterial and fungal proteins such as toxins, virulence factors, adhesins, and surface proteins have parallel beta-helices which play important role in human infectious disease [33]. Therefore, BetaWrap program [23] was used to predict the super secondary structural motif in primary amino acid sequences of 25 antigens. A total 9 candidate antigens (numbers 2–6, 9, 11, 13, and 24) were predicted to contain right-handed parallel beta-helix.

Besides, heterologous immunity may exist to cross-reactive epitopes in other strains of the same organism. Thus, we identified the potential orthologs in the available* Leishmania* genomes annotations using OrthoMCL database [24] through BLASTP homology prediction program. All the selected 25 candidate antigens showed orthologs in other related species, namely*, L. braziliensis*,* L. infantum,* and* L. mexicana* except antigen number 11. One of the greatest barriers in vaccine development is the possibility that a particular vaccine may cross-react between host and parasite antigens [34]. Thus, vaccine candidates showing sequence similarity with the hosts (e.g., human or mouse) proteins are likely to cause autoimmunity in the host and should be discarded to avoid potential autoimmunity. Out of 25 antigens, 3 (numbers 3, 9, and 18) showed orthologs in human as well as mouse.

### 3.2. Epitope Based Analysis of the Selected Antigens Using NetCTL

For elicitation of T cell responses, the subcellular location and function of target protein are less important than the presence of appropriate MHC binding epitopes in the protein sequences [35]. In past 15 years, significant efforts have been made toward generation of procedures/algorithms for accurate prediction of MHC binding affinity and T cell epitopes. Utilizing the clustering method, majority of HLA molecules have been classified in relatively few HLA supertypes on the basis of their peptide binding specificities [36, 37]. One approach to identifying targets of CTL responses in an antigen is based on prediction of high affinity MHC class I restricted T cell epitopes using computerized algorithms [38]. It is also demonstrated that peptides that possess* in vitro* binding affinity (IC_50_) values of ≤ 500 nM are more immunogenic* in vivo* [39].

Thus, in the current study, the immunogenicity screening was limited to the predicted peptides that were able to bind HLA class I supertypes, with binding affinities (IC_50_) ≤500 nM [25]. Furthermore, it is important to consider whether each MHC binding peptide is being correctly processed from the native antigen and subsequently displayed on the surface of antigen presenting cells. At present, it is possible to predict the naturally processed peptides using NetCTL algorithm above a combined epitope processing score of 0.75, which includes predictions of proteasomal cleavage, TAP binding, and HLA binding [26]. Thus, in order to identify candidate CD8+ T cell epitopes, 25 candidate antigens selected from* L. major* were screened using NetCTL.

**Figure 2:**
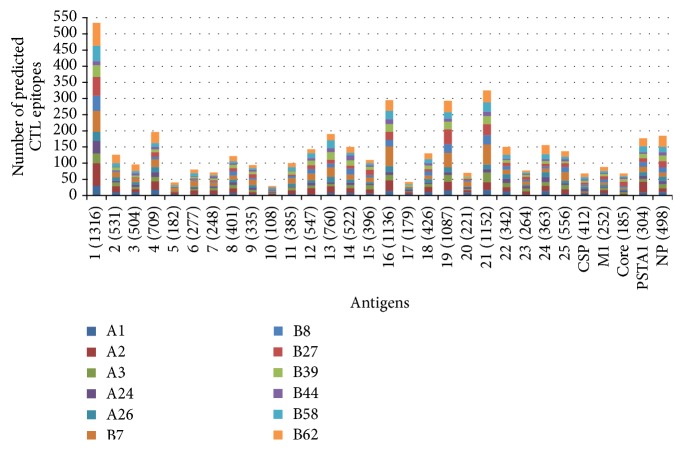
Graphical representation of CTL epitopes predicted by NetCTL for the 25 potential* L. major* antigens and 5 control antigens (CSP, M1, core, PSTA1, and NP), which bind with at least one allele in the HLA-A (A1, A2, A3, A24, and A26) and HLA-B (B7, B8, B27, B39, B44, B58, and B62) supertypes.

The tool NetCTL1.2 provides a comprehensive prediction about epitopes binding to the 12 different HLA class I supertypes: HLA-A1, HLA-A2, HLA-A3, HLA-A24, and HLA-A26 and HLA-B7, HLA-B8, HLA-B27, HLA-B39, HLA-B44, HLA-B58, and HLA-B62. A total of 3756 putative CTL epitopes were predicted, including 1373 HLA-A (230-A1, 429-A2, 269-A3, 207-A24, and 238-A26) and 2383 HLA-B (542-B7, 283-B8, 318-B27, 288-B39, 157-B44, 304-B58, and 491-B62) supertype binding peptides (Figure 2). Predictions for the 5 control antigens showed that CSP had 28-HLA-A, 40-HLA-B, M1 had 33-HLA-A, 55-HLA-B, core had 21-HLA-A, 48-HLA-B, PSTA1 had 83-HLA-A, 104-HLA-B, and NP had 59-HLA-A, 126-HLA-B restricted CTL epitopes. Apart from this, their experimentally validated CTL epitopes were also predicted by NetCTL ranked in top five. From the analysis, antigen number 1 was found to have largest number of predicted CTL epitopes for HLA-A and HLA-B supertypes while antigens numbers 4, 16, 19, and 21 had higher number of CTL epitopes for HLA-A supertypes and antigens numbers 13, 16, 19, and 21 had higher number of CTL epitopes for HLA-B supertypes in comparison to the control antigens. Overall, test antigen number 1 showed highest number of supertype epitopes and was thus predicted as best antigen. From among the predicted CTL epitopes, the epitopes which displayed the top processing score for the respective MHC supertypes are presented in Tables 2 and 3. These 624 potential CTL epitopes were also checked for their potential similarity with human proteins, using Human Protein Reference Database (http://www.hprd.org/). However, none of the epitopes were found similar to any human proteins [40].

### 3.3. Selection of Optimal Epitopes Set Based on Population Coverage Analysis

MHC is highly polymorphic; hence each individual possesses a set of MHC molecules of differing specificities; that is, different patients typically bind different repertoires of peptides. Thus, a crucial step in the design of effective peptide based vaccine is the selection of the good epitopes set which yields the best immune response in a given population or individual. Furthermore, the frequency of an MHC allele to occur within the target human population directly affects the allele's contribution to the overall immunogenicity. Sette et al. have also demonstrated a correlation between immunogenicity and MHC class I binding affinity [39]. It is therefore reasonable to use MHC class I binding affinity prediction methods for calculation of the overall immunogenicity [40]. Hence, the present study employed OptiTope (using BIMAS method [41]) to determine the optimal set of epitopes from the selected epitopes, which calculate the best immune response in the susceptible target populations of cutaneous leishmaniasis, namely, Southwest Asia, North Africa, and South America.

Initially, the top scoring epitope sets predicted using NetCTL from each of the 25 candidate antigens were tried to optimize by OptiTope and screened the BIMAS based HLA nonbinders (negative) for the target populations. Out of these, 10 epitope sets (from antigen numbers 1, 2, 4, 5, 6, 8, 15, 21, 22, and 25) that were predicted HLA binders (positive) are clubbed together to form a set of 120 candidate epitopes. However, when this combined epitope set was further optimized for the three different target populations, no optimization solution was obtained for any population. Therefore, epitope set of antigen number 21 was randomly excluded from the combined set and got the optimized results with the 108 combined epitopes set (derived from the 9 positive antigens) for the different target populations.

For the target population of Southwest Asia, out of the 108 candidate epitopes set, OptiTope selected a subset of 60 epitopes restricted by the 19 MHC class I alleles covering 96.58 % human population. The most immunogenic epitope, 127SLWSLLAGV, from antigen number 1, was found to bind with allele HLA-A^*∗*^0201, contributing 7%, while the least immunogenic epitope, 246KSSALAHKL, from antigen number 25, contributed < 1% to the overall immunogenicity (Table 4).

Similarly, for the target population of North Africa, out of the 108 candidate epitopes' set, OptiTope selected a subset of 45 epitopes restricted by the 13 MHC class I alleles covering 88.48 % human population. Here again, the epitope 127SLWSLLAGV, from antigen number 1, was most immunogenic, covered the allele HLA-A^*∗*^0201, and contributes 9%, and the least immunogenic epitope, 4LLPRLFLAF, from antigen number 8, contributed <1% to the overall immunogenicity (Table 5).

Also, for the target population of South America, out of the 108 candidate epitopes' set, OptiTope selected a subset of 34 epitopes restricted by the 9 MHC class I alleles covering 98.97 % human population. For a third time, the epitope 127SLWSLLAGV, from antigen number 1, which binds to the allele HLA-A^*∗*^0201, was predicted most immunogenic and contributed 10%, while the least immunogenic epitope, 127SIMSLQIRY, from antigen number 8, was found to contribute <1% to the overall immunogenicity (Table 6).

Thus, overall, it was found that 6 antigens (numbers 1, 4, 13, 16, 19, and 21) had larger number of predicted CTL epitopes as compared to control antigens which could be tested* in vivo* for validation. Similarly, the epitope 127SLWSLLAGV, from antigen number 1, binds to HLA-A^*∗*^0201 molecule and was predicted as most immunogenic for all the three populations' susceptibility to leishmaniasis [42, 43]. Hence, these antigens/peptides may be considered as suitable candidates for vaccine and diagnostics design [44]. Further, these protective epitopes conform to the anchor-based MHC binding motifs' concept used for T cell epitope identification by many researchers such as Sette et al. [45] and Rötzschke et al. [46].

## 4. Conclusions

The current study aimed to mine* L. major* genome for antigens selection and characterization as vaccine components based on criteria such as presence of transmembrane domains and orthologs analysis. Furthermore, the immunogenic epitopes predicted from these antigens can be analyzed for HLA-supertype binding and optimization of good vaccine epitopes against susceptible human populations to* L. major* infection. In light of the results obtained, it can be concluded that the combined use of reverse vaccinology and immunoinformatics along with* in vitro*/*in vivo* validation strategies has emerged as the most promising approach in designing successful vaccine against tropical diseases.

In future, it would be helpful to use modeling and simulation system where critical experiments may be performed in a computer in order to predict the effects of experimental modifications on the immune system and thus offer a criterion for the selection of the most likely meaningful experimental tests to be conducted* in vivo* or* in vitro*.

## Figures and Tables

**Table 1:** List of predicted 25 *L. major *candidate antigens and 5 control antigens along with their prediction probabilities using SOLpro and TMHMM program.

Antigen number	GeneDB ID/UniProt accession number	Number of amino acids	Function	Protein solubility upon overexpression prediction probabilities	Number of TM regions
1	LmjF.03.0550	1316	Hypothetical protein, conserved	0.947197 Insoluble	5
2	LmjF.04.0130	531	Hypothetical protein, conserved in *Leishmania*	0.814123 Insoluble	0
3	LmjF.04.0180	504	Surface antigen-like protein	0.819598 Soluble	1
4	LmjF.04.0190	709	Surface antigen-like protein	0.931571 Insoluble	1
5	LmjF.04.0200	182	Surface antigen-like protein	0.769865 Soluble	0
6	LmjF.04.0210	277	Surface antigen-like protein	0.913911 Insoluble	2
7	LmjF.04.0910	248	Hypothetical protein, conserved	0.586919 Soluble	0
8	LmjF.06.0380	401	Hypothetical protein, conserved	0.76955 Insoluble	0
9	LmjF.09.0850	335	Ras family protein-like protein	0.612666 Insoluble	0
10	LmjF.12.0710	108	Hypothetical protein, conserved	0.556695 Insoluble	1
11	LmjF.12.1000	385	Promastigote surface antigen protein 2, PSA2	0.802331 Insoluble	2
12	LmjF.12.0870	547	Surface antigen protein 2, putative	0.696157 Soluble	2
13	LmjF.12.0740	760	Surface antigen protein, putative	0.534869 Soluble	2
14	LmjF.13.0480	522	Hypothetical protein, conserved	0.785913 Soluble	0
15	LmjF.14.0770	396	Hypothetical protein, unknown function	0.705749 Insoluble	0
16	LmjF.16.0620	1136	Hypothetical protein, unknown function	0.682847 Soluble	0
17	LmjF.17.1350	179	Hypothetical protein, conserved	0.625526 Insoluble	1
18	LmjF.22.0470	426	Hypothetical protein, conserved	0.743393 Soluble	0
19	LmjF.22.1260	1087	Hypothetical protein, conserved	0.689942 Insoluble	0
20	LmjF.23.0225	221	Hypothetical protein, conserved	0.870557 Insoluble	1
21	LmjF.24.1520	1152	Hypothetical protein, conserved	0.588925 Insoluble	0
22	LmjF.26.0340	342	Hypothetical protein, conserved	0.671966 Insoluble	1
23	LmjF.28.2565	264	Hypothetical protein, conserved	0.607781 Insoluble	0
24	LmjF.32.0510	363	Hypothetical protein, conserved	0.825034 Insoluble	2
25	LmjF.33.1890	556	Hypothetical protein, conserved	0.558399 Insoluble	1
CSP	P02893	412	Circumsporozoite protein	0.857803 Soluble	0
M1	P36347	252	Matrix protein 1	0.655264 Insoluble	0
NP	P03466	498	Nucleoprotein	0.528252 Soluble	0
Core	CAA59535.1	185	HBV core	0.816707 Insoluble	0
PSTA1	P9WG10	304	Phosphate transport system permease protein 1	0.654434 Insoluble	6

**Table 2:** NetCTL predicted top scoring CTL epitopes along with start position and processing score for the HLA-A supertypes in 25 *L. major* candidate antigens.

Antigens	A1	A2	A3	A24	A26
1	NTDNFFFML (228: 2.3073)	SLWSLLAGV (127: 1.4042)	NLAAGQSLK (489: 1.4921)	LYLLLPFLL (19: 1.9727)	YTISLNPLL (512: 1.6485)

2	MSSTSFDDY (38: 3.3876)	ALVSINVPL (224: 1.3414)	SLFRVANCK (238: 1.4763)	VWCTVPDCI (421: 1.4489)	SVVDPMQNY (409: 2.3630)

3	CTQCAPNYY (308: 2.8811)	LLTSFAMHL (495: 1.0227)	SSYSCVSQK (469: 1.6062)	GYAKDSNGF (175: 1.5045)	YVVDSYDGL (351: 1.4847)

4	FIDANTAIY (122: 3.1585)	MLPDMTCSL (390: 1.4053)	SSYSCVSQK (674: 1.6062)	GYIVVDKYF (93: 1.4189)	YVVDSYDGL (556: 1.4847)

5	TTSTTTNTV (68: 1.4293)	LMAAMLVAV (7: 1.2898)	TMPTAPSSK (43: 1.1211)	GYMPTASFK (142: 0.8997)	ETASTTSTT (64: 0.5879)

6	VSAQTIDDY (32: 2.4784)	YLCDRTTAA (123: 1.1971)	VSYTCIPRK (241: 1.5973)	GYPNINTYL (116: 1.1822)	QTIDDYPPV (35: 1.4284)

7	TAAVKPLSY (18: 1.8789)	TLASHPHYL (208: 1.2381)	RVAEFLVVK (198: 1.3425)	HYLHEANVF (214: 1.6364)	EVPICSLEF (185: 1.0192)

8	SIMSLQIRY (127: 1.1734)	FLFSPTDTL (12: 1.3828)	RIKRNWQKK (38: 1.3737)	IFMRLEDTI (185: 1.4289)	SIIEKATRY (200: 2.2008)

9	YREILNEFY (162: 1.6879)	FVAKYIPTI (96: 1.3345)	LMMSCWSAR (3: 1.1799)	LYTPALPPF (17: 1.7705)	EVIEDLVVW (321: 1.7072)

10	SASNHKEFY (11: 1.8707)	RMDVIGATV (52: 1.1546)	EFYIYYLAK (17: 0.7897)	QWTRRMHLI (29: 1.3589)	SASNHKEFY (11: 0.9535)

11	LTDEKTCLK (346: 1.7725)	FLTDEKTCL (345: 1.2608)	QAFGRAIPK (51: 1.2646)	TYAGTLPEM (90: 1.0075)	YVSGISPTY (83: 1.6128)

12	LTDERTCLV (508: 2.3650)	FLTDERTCL (507: 1.2308)	RIQQLVLRK (230: 1.3906)	LYIWNMPLL (112: 1.9046)	TTITSTTKL (445: 1.1733)

13	LTDERTCLV (721: 2.3650)	MLSAENLQL (469: 1.2642)	KSLTNLYLK (422: 1.3473)	EWSRVTSLL (200: 1.6263)	EMKSLTNLY (396: 1.8138)

14	DLEEEVEEY (115: 1.8614)	TLLEQYASL (292: 1.2313)	LLEQYASLK (293: 1.3401)	SFPPSPSLL (2: 1.4148)	QVKELKVSY (182: 1.2390)

15	QTRVHPGLY (116: 2.0525)	YLLDGDQLI (71: 1.4042)	RSAPHHSRR (226: 1.3101)	LFGAFLFAF (388: 1.2731)	DVKESNAHV (48: 1.1458)

16	LVDTTAWRY (1027: 3.6396)	MLWETVAAL (290: 1.3564	RTATARLHK (248: 1.5070)	LFQRVLAPI (982: 1.1324)	EAQDHSCFY (628: 1.9956)

17	KADTYVEEF (82: 1.3354)	VLAVVVLLV (10: 1.1790)	HLRGAATGK (74: 1.4033)	DMATVFAYF (151: 1.2555)	STVRLLVSF (163: 1.4330)

18	ATSNAASRY (119: 3.2566)	TMADVLLYA (135: 1.2997)	ATMADVLLY (134: 1.3320)	QFLINSSSI (2: 1.2187)	ATMADVLLY (134: 1.7501)

19	FTSGEISFY (21: 3.4722)	YMNLISQSI (1056: 1.2709)	LLYCRESRK (1039: 1.6445)	AYLRELFPV (702: 1.3356)	FTSGEISFY (21: 2.3098)

20	NTTTAVRGY (27: 1.7205)	ILMWSFAAL (204: 1.3025)	ALFVVMAMY (211: 1.2263)	NWWILMWSF (201: 1.5092)	NTTTAVRGY (27: 1.5857)

21	LASLLSSKY (1096: 1.7120)	ALARYPLPV (58: 1.3281)	ALASLLSSK (1095: 1.5764)	VYILLTEFL (1138: 1.6493)	HVARQLASY (980: 2.0702)

22	YMDPGAAGY (201: 3.0231)	TLFPIDVTV (220: 1.3367)	ALYTSIPVR (288: 1.4227)	LFLLVIYAF (35: 1.7091)	EAAHFLMAY (155: 2.1321)

23	CTGSSPSVY (8: 2.2878)	VLIDYLLSM (252: 1.4781)	TLASSAAVK (184: 1.5850)	VYFTLPTAV (15: 1.3233)	VLIDYLLSM (252: 1.3831)

24	LTAPVYMQY (106: 3.2540)	LMFSLSQSL (98: 1.2637)	RLTPFFQNY (115: 1.2825)	LYRIDGTLI (181: 1.3091)	ATGDQVSGY (155: 1.9446)

25	ISDTQVLLA (264: 1.5818)	VLVGVVLGV (539: 1.2310)	LVHAGIAGK (486: 1.3287)	AYFVVPLEM (356: 1.1485)	STVLRLFSF (24: 1.4346)

**(a):** 

Antigens	B7	B8	B27	B39
1	APALYTISL (508: 1.7598)	PLRWRFRAV (957: 2.1122)	RRAKRGIQK (908: 1.8727)	YQLTGTPVL (439: 2.2884)

2	TPSSARLSM (83: 1.7256)	FPISKGAAL (203: 2.2300)	VRVDTQSSF (195: 1.4558)	SFDDYTMVL (42: 2.2280)

3	APNYYLTPL (312: 1.7308)	YSLWVAAAV (486: 1.0021)	SRAILIAVL (3: 1.3691)	SRAILIAVL (3: 1.8971)

4	APNYYLTPL (517: 1.7308)	FVRVWDRSL (215: 1.8335)	LRVSHSSVK (224: 1.3540)	VRAPFTIQL (155: 1.5796)

5	APAHGSVSL (33: 1.7709)	VKHLLMAAM (3: 1.4045)	DRLGQCMVV (109: 0.5955)	KHLLMAAML (4: 1.0764)

6	SPTPLLAAL (256: 1.6602)	NPHKRGAAA (9: 1.8533)	KRGAAAVLL (12: 1.3311)	HKRGAAAVL (11: 1.2133)

7	APSPCVPPL (175: 1.5944)	AAYRSYAAV (144: 0.9391)	MQVLLGADF (237: 0.8288)	SHDGKHVIL (31: 2.6870)

8	LPRLFLAFL (5: 1.6144)	LLPRLFLAF (4: 1.5126)	GRIKRNWQK (37: 1.4947)	IHPERTVAL (294: 2.2073)

9	SARARTLSL (9: 1.8057)	YPRIKLLVI (69: 2.0337)	ARARTLSLY (10: 1.2183)	YEAAQGVLL (170: 1.5760)

10	QPTTFKNPI (80: 1.1417)	RMHLIGTAV (33: 1.4672)	KQWTRRMHL (28: 1.5430)	HKEFYIYYL (15: 1.4474)

11	TPRTTTEPL (274: 1.7685)	MSKARSLQL (181: 1.3726)	RRLVLAATL (6: 1.9702)	QRTNTLAVL (42: 1.4593)

12	MPYLRGVSL (300: 1.8440)	MPYLRGVSL (300: 2.1953)	RRLVLAATL (6: 1.9702)	YRHVMIREL (104: 1.7846)

13	MPRLRLVGL (493: 1.7353)	MPRLRLVGL (493: 2.2824)	RRLVLAATL (6: 1.9702)	TAAQRTHTL (39: 1.5626)

14	MPAPPLNPF (414: 1.6955)	KEKERHKAV (94: 1.8613)	RRLMPAPPL (411: 1.7237)	YESNTVSAL (317: 2.1937)

15	TPRIPLDSL (187: 1.5747)	SSHRKHKAM (162: 1.6542)	RRMRAGSSH (250: 1.6225)	AHAPQNAAL (138: 2.5007)

16	MPRKRGRPL (237: 1.8778)	MPRKRGRPL (237: 2.3735)	RRTLQAQQL (546: 1.5424)	DHAQGVAAL (877: 2.1234)

17	VPHHPGGDV (134: 0.9985)	YVEEFYQAA (86: 0.7269)	KRVMAPSDR (37: 1.1717)	FHDPSTVRL (159: 2.8191)

18	HPSGAAVAI (409: 1.4883)	RLYVEDMVL (284: 1.2511)	RRAEKEKAK (215: 1.7438)	NHSAHTEVL (147: 2.1507)

19	KPSAVMTAF (919: 1.8285)	EPSRRTVQF (667: 1.7157)	RRWAAQNTF (77: 2.1177)	FRVDGADAL (675: 2.1322)

20	AARQRIMTM (156: 1.6084)	ILMWSFAAL (204: 1.7177)	RRAPTGLYE (71: 0.9324)	QLDDNWWIL (197: 1.3196)

21	APAAPHSPL (153: 1.7675)	ELRRRGQEV (1113: 1.9415)	RRLLAASPF (877: 2.0769)	NQATTSLAL (504: 1.9553)

22	YPAHRSKIV (163: 1.5878)	DAQVRQTAL (281: 1.8406)	QRRDVVIGM (75: 1.3697)	SHLVSVDKL (331: 1.5360)

23	TPRVGCSVA (63: 1.2802)	FFRRYTRVF (46: 2.1834)	RRYTRVFPA (48: 1.7379)	YFTLPTAVL (16: 1.6593)

24	SPLSVSAVF (27: 1.5060)	YMQYRLTPF (111: 2.0206)	FRYDHINSY (61: 1.7109)	YASQKFVQL (311: 1.2960)

25	RPRLFARAI (256: 1.7812)	RLPRRLQAM (301: 2.1397)	RRLLVHAGI (483: 1.8779)	HSEAATSSL (72: 1.7315)

**(b):** 

Antigens	B44	B58	B62
1	RELQSVYLL (1293: 1.9593)	GTFAAPLRW (952: 2.0156)	SQQETSSLY (802: 1.4851)

2	VESGALFSF (276: 1.5005)	VSGGSTVSF (299: 1.2478)	LVVDASSLF (232: 1.2364)

3	AECDTGYSL (383: 1.7888)	SAAAPYSLW (481: 1.7104)	VINSAAAPY (478: 1.2841)

4	AECDTGYSL (588: 1.7888)	SAAAPYSLW (686: 1.7104)	AMKDPYTNY (355: 1.5075)

5	PEQSKNAAL (153: 0.9583)	AMASDASSW (20: 1.2946)	SGYMPTASF (141: 1.1587)

6	CESGYALTV (233: 1.5835)	VAATVACVM (269: 1.3253)	SGYALTVSY (235: 1.2956)

7	HEANVFGDL (217: 1.4597)	ITLASHPHY (207: 1.8535)	MQVLLGADF (237: 1.4107)

8	EEHKFHEQL (234: 1.7745)	STQPPVSSW (375: 1.3179)	QFIQGRCPY (279: 1.2654)

9	YEAAQGVLL (170: 1.8058)	QSFAALQSW (186: 1.9574)	REHGCAAYY (301: 1.0743)

10	KEFYIYYLA (16: 1.2817)	TAVGVAICW (62: 1.8196)	RMHLIGTAV (33: 1.0529)

11	PEWGSMTSL (152: 1.5995)	ISGSVPPEW (146: 1.9830)	YVSGISPTY (83: 1.4703)

12	LEGLTSLTL (131: 1.7189)	ITGPLPPQW (241: 1.9075)	YVRVISTTY (83: 1.4798)

13	SEMKSLTSL (323: 1.9322)	GSLPSEWSW (171: 1.9009)	TQVSGTLPL (336: 1.2966)

14	GEFSDIRQL (25: 1.9010)	AAVADAEVW (458: 1.6950)	QVKELKVSY (182: 1.3565)

15	AQTRVHPGL (115: 0.9806)	MSLFVSTLF (381: 1.5159)	FVSTLFGAF (384: 1.2037)

16	GEARNPHRL (724: 1.7322)	AASAPSFQW (501: 1.9979)	RLAAEAQGF (516: 1.3449)

17	EFYQAAGHL (89: 0.5235)	KADTYVEEF (82: 1.5709)	QDMATVFAY (150: 1.1423)

18	GEDEEQVSL (71: 1.8499)	SAAKAQVSY (196: 1.3995)	IVSGLVESY (299: 1.3837)

19	AEHRRGTQL (978: 1.4654)	CASTATHVF (601: 1.7234)	MMSQSLSTY (1: 1.4755)

20	AEMQRNIDR (60: 0.5443)	LMWSFAALF (205: 1.0739)	RGYTRGIPY (33: 1.3338)

21	TESVQFLKL (968: 1.5363)	LTSAINQFW (140: 1.8100)	SMMLPAGDF (797: 1.4153)

22	FEAPLGEML (132: 1.6939)	VAFACYFLF (26: 1.6804)	VLFTDGTPY (57: 1.4757)

23	QEAKARTTV (193: 1.3216)	TGSSPSVYF (9: 1.0291)	AMHDDQLRF (38: 1.2996)

24	KEPGHKIPL (256: 1.9127)	LTAPVYMQY (106: 1.7822)	SQSLTAPVY (103: 1.4574)

25	REWYSADVL (525: 1.8122)	KSSALAHKL (246: 1.5564)	RVVKQSLCF (128: 1.1964)

**Table 4:** The distribution of fractional immunogenicity for the 60 optimal epitopes against the population of Southwest Asia.

S. number	Epitope	Fraction of overall immunogenicity	Covered alleles
1	*SLWSLLAGV*	0.07	A^*∗*^0201
2	LPRLFLAFL	0.06	B^*∗*^0702 B^*∗*^0801 B^*∗*^3501 Cw^*∗*^0401 Cw^*∗*^0602
3	YLLDGDQLI	0.05	A^*∗*^0201 A^*∗*^0205
4	GYPNINTYL	0.05	A^*∗*^2402 Cw^*∗*^0401
5	LYLLLPFLL	0.05	A^*∗*^2402 Cw^*∗*^0401
6	YMDPGAAGY	0.05	A^*∗*^0101
7	TPRIPLDSL	0.05	B^*∗*^0702 B^*∗*^0801 B^*∗*^3501 Cw^*∗*^0401
8	SPTPLLAAL	0.04	B^*∗*^0702 B^*∗*^3501 Cw^*∗*^0401 Cw^*∗*^0602 Cw^*∗*^0702
9	SFDDYTMVL	0.04	A^*∗*^2402 B^*∗*^3801 Cw^*∗*^0401
10	GYIVVDKYF	0.03	A^*∗*^2402
11	VLVGVVLGV	0.03	A^*∗*^0201
12	TLFPIDVTV	0.03	A^*∗*^0201
13	FIDANTAIY	0.03	A^*∗*^0101
14	FLFSPTDTL	0.02	A^*∗*^0201 A^*∗*^0205
15	LMAAMLVAV	0.02	A^*∗*^0201
16	APNYYLTPL	0.02	B^*∗*^0702 B^*∗*^3501 Cw^*∗*^0401
17	SLFRVANCK	0.02	A^*∗*^0301
18	LFLLVIYAF	0.02	A^*∗*^2402 Cw^*∗*^0401
19	MLPDMTCSL	0.02	A^*∗*^0201 A^*∗*^0205
20	ELRRRGQEV	0.02	B^*∗*^0801
21	LFGAFLFAF	0.02	Cw^*∗*^0401 Cw^*∗*^0702
22	LVHAGIAGK	0.02	A^*∗*^1101 A^*∗*^6801
23	FPISKGAAL	0.02	B^*∗*^0702 B^*∗*^0801 B^*∗*^3501
24	RPRLFARAI	0.01	B^*∗*^0702 B^*∗*^3501 B^*∗*^5101
25	STQPPVSSW	0.01	B^*∗*^5801
26	GYMPTASFK	0.01	A^*∗*^1101
27	APAAPHSPL	0.01	B^*∗*^0702 B^*∗*^3501
28	LTSAINQFW	0.01	B^*∗*^5801
29	IHPERTVAL	0.01	B^*∗*^3801
30	VESGALFSF	0.01	B^*∗*^4403
31	YQLTGTPVL	0.01	A^*∗*^0205 B^*∗*^5201
32	GTFAAPLRW	0.01	B^*∗*^5801
33	YVVDSYDGL	0.01	A^*∗*^0205
34	APALYTISL	0.01	B^*∗*^0702 B^*∗*^3501
35	APAHGSVSL	0.01	B^*∗*^0702 B^*∗*^3501
36	AYFVVPLEM	0.01	A^*∗*^2402
37	KHLLMAAML	0.01	B^*∗*^3801 Cw^*∗*^0602
38	FVRVWDRSL	0.01	B^*∗*^0702 B^*∗*^0801
39	AHAPQNAAL	0.01	B^*∗*^3801
40	ALYTSIPVR	0.01	A^*∗*^0301
41	MSLFVSTLF	0.01	B^*∗*^5801
42	TPSSARLSM	0.01	B^*∗*^0702 B^*∗*^3501
43	STVLRLFSF	0.01	B^*∗*^5801
44	VSGGSTVSF	0.01	B^*∗*^5801
45	SAAAPYSLW	0.01	B^*∗*^5801
46	NLAAGQSLK	<0.01	A^*∗*^0301
47	TMPTAPSSK	<0.01	A^*∗*^0301
48	CESGYALTV	<0.01	B^*∗*^4006
49	VSAQTIDDY	<0.01	Cw^*∗*^0702
50	YLCDRTTAA	<0.01	A^*∗*^0201
51	RELQSVYLL	<0.01	B^*∗*^4006 B^*∗*^4403
52	RIKRNWQKK	<0.01	A^*∗*^1101
53	HVARQLASY	<0.01	Cw^*∗*^0702
54	SVVDPMQNY	<0.01	Cw^*∗*^0702
55	AMKDPYTNY	<0.01	Cw^*∗*^0702
56	LLPRLFLAF	<0.01	A^*∗*^0301
57	REWYSADVL	<0.01	B^*∗*^4006
58	YTISLNPLL	<0.01	Cw^*∗*^0602
59	VRAPFTIQL	<0.01	Cw^*∗*^0602
60	KSSALAHKL	<0.01	Cw^*∗*^0602

**Table 5:** The distribution of fractional immunogenicity for the 45 optimal epitopes against the population of North Africa.

S. number	Epitope	Fraction of overall immunogenicity	Covered alleles
1	*SLWSLLAGV*	0.09	A^*∗*^0201
2	YMDPGAAGY	0.09	A^*∗*^0101
3	YLLDGDQLI	0.07	A^*∗*^0201
4	TPRIPLDSL	0.05	B^*∗*^0702 B^*∗*^0801 B^*∗*^3501
5	LPRLFLAFL	0.05	B^*∗*^0702 B^*∗*^0801 B^*∗*^3501
6	FIDANTAIY	0.05	A^*∗*^0101
7	VESGALFSF	0.04	B^*∗*^4403
8	VLVGVVLGV	0.04	A^*∗*^0201
9	TLFPIDVTV	0.04	A^*∗*^0201
10	ELRRRGQEV	0.04	B^*∗*^0801
11	GYPNINTYL	0.03	A^*∗*^2402
12	LYLLLPFLL	0.03	A^*∗*^2402
13	LMAAMLVAV	0.03	A^*∗*^0201
14	FPISKGAAL	0.03	B^*∗*^0702 B^*∗*^0801 B^*∗*^3501
15	FLFSPTDTL	0.03	A^*∗*^0201
16	GYIVVDKYF	0.03	A^*∗*^2402
17	MLPDMTCSL	0.02	A^*∗*^0201
18	SLFRVANCK	0.02	A^*∗*^0301
19	APAAPHSPL	0.02	B^*∗*^0702 B^*∗*^3501
20	RPRLFARAI	0.01	B^*∗*^0702 B^*∗*^3501 B^*∗*^5101
21	ALYTSIPVR	0.01	A^*∗*^0301 A^*∗*^3101
22	APALYTISL	0.01	B^*∗*^0702 B^*∗*^3501
23	APNYYLTPL	0.01	B^*∗*^0702 B^*∗*^3501
24	APAHGSVSL	0.01	B^*∗*^0702 B^*∗*^3501
25	FVRVWDRSL	0.01	B^*∗*^0702 B^*∗*^0801
26	STQPPVSSW	0.01	B^*∗*^5801
27	LVHAGIAGK	0.01	A^*∗*^1101 A^*∗*^6801
28	LTSAINQFW	0.01	B^*∗*^5801
29	GTFAAPLRW	0.01	B^*∗*^5801
30	AYFVVPLEM	0.01	A^*∗*^2402
31	SPTPLLAAL	0.01	B^*∗*^0702 B^*∗*^3501
32	TPSSARLSM	0.01	B^*∗*^0702 B^*∗*^3501
33	MSLFVSTLF	0.01	B^*∗*^5801
34	GYMPTASFK	0.01	A^*∗*^1101
35	STVLRLFSF	0.01	B^*∗*^5801
36	YLCDRTTAA	0.01	A^*∗*^0201
37	NLAAGQSLK	<0.01	A^*∗*^0301
38	TMPTAPSSK	<0.01	A^*∗*^0301
39	VSGGSTVSF	<0.01	B^*∗*^5801
40	SAAAPYSLW	<0.01	B^*∗*^5801
41	RELQSVYLL	<0.01	B^*∗*^4403
42	SFDDYTMVL	<0.01	A^*∗*^2402
43	LFLLVIYAF	<0.01	A^*∗*^2402
44	RIKRNWQKK	<0.01	A^*∗*^1101
45	LLPRLFLAF	<0.01	A^*∗*^0301

**Table 6:** The distribution of fractional immunogenicity for the 34 optimal epitopes against the population of South America.

S. number	Epitope	Fraction of overall immunogenicity	Covered alleles
1	*SLWSLLAGV*	0.1	A^*∗*^0201
2	ALYTSIPVR	0.1	A^*∗*^3101
3	GYPNINTYL	0.08	A^*∗*^2402 Cw^*∗*^0401
4	YLLDGDQLI	0.08	A^*∗*^0201
5	LYLLLPFLL	0.08	A^*∗*^2402 Cw^*∗*^0401
6	GYIVVDKYF	0.05	A^*∗*^2402
7	VLVGVVLGV	0.05	A^*∗*^0201
8	TLFPIDVTV	0.04	A^*∗*^0201
9	SFDDYTMVL	0.04	A^*∗*^2402 B^*∗*^3901 Cw^*∗*^0401
10	VSAQTIDDY	0.04	Cw^*∗*^0702
11	SPTPLLAAL	0.03	Cw^*∗*^0401 Cw^*∗*^0702
12	LMAAMLVAV	0.03	A^*∗*^0201
13	FLFSPTDTL	0.03	A^*∗*^0201
14	HVARQLASY	0.03	Cw^*∗*^0702
15	MLPDMTCSL	0.02	A^*∗*^0201
16	LFLLVIYAF	0.02	A^*∗*^2402 Cw^*∗*^0401
17	LFGAFLFAF	0.02	Cw^*∗*^0401 Cw^*∗*^0702
18	AYFVVPLEM	0.02	A^*∗*^2402
19	KHLLMAAML	0.02	B^*∗*^3901
20	IHPERTVAL	0.02	B^*∗*^3901
21	LPRLFLAFL	0.02	Cw^*∗*^0401
22	TPRIPLDSL	0.01	Cw^*∗*^0401
23	LVHAGIAGK	0.01	A^*∗*^6801
24	AHAPQNAAL	0.01	B^*∗*^3901
25	APNYYLTPL	0.01	Cw^*∗*^0401
26	SVVDPMQNY	0.01	Cw^*∗*^0702
27	AMKDPYTNY	0.01	Cw^*∗*^0702
28	YLCDRTTAA	0.01	A^*∗*^0201
29	LLPRLFLAF	0.01	B^*∗*^1501
30	SQQETSSLY	0.01	B^*∗*^1501
31	YQLTGTPVL	<0.01	B^*∗*^5201
32	VRAPFTIQL	<0.01	B^*∗*^3901
33	KRGAAAVLL	<0.01	B^*∗*^3901
34	SIMSLQIRY	<0.01	B^*∗*^1501
